# IFNβ absence compensates for LAT functions in latency reactivation and T cell exhaustion

**DOI:** 10.1128/jvi.00374-25

**Published:** 2025-05-12

**Authors:** Shaohui Wang, Ujjaldeep Jaggi, Jay J. Oh, Homayon Ghiasi

**Affiliations:** 1 Center for Neurobiology and Vaccine Development, Ophthalmology Research, Department of Surgery, Cedars-Sinai Medical Center 22494, Los Angeles, California, USA; St. Jude Children's Research Hospital, Memphis, Tennessee, USA

**Keywords:** knockout, type 1 interferon, IFNβ, ocular infection, eye disease, virus replication, latency, reactivation, CD8, PD-1, exhaustion

## Abstract

Type I interferons (IFNs) generate strong antiviral immunity following viral infection in mice and humans. These type I IFNs are encoded by at least 14 IFNα genes and a single IFNβ gene. We showed that the absence of one of the IFNα genes (IFNα2A^-/-^) affected latency levels in herpes simplex virus type 1 (HSV-1) ocularly infected mice but not in infected control mice, and the absence of IFNα2A did not affect viral reactivation in ocularly infected mice. Since the role of IFNβ in HSV-1 latency reactivation and the potential effect of latency-associated transcript (LAT) on IFNβ activity is not known, we ocularly infected IFNβ^-/-^ mice with different doses of LAT-plus [LAT(+)] and LAT-minus [LAT(-)] viruses. Wild-type (WT) control mice and IFNβ^-/-^ mice were infected similarly. Virus titers in the eye, viral and cellular transcripts in the eye and trigeminal ganglia (TG) of infected mice on days 3 and 5 post-infection, eye disease, survival, latency-reactivation levels, and T cell exhaustion were measured in latently infected mice. Virus replication and viral and cellular transcripts in the eye of infected IFNβ^-/-^ mice were similar to those in WT mice, while eye disease and survival in IFNβ^-/-^ mice differed significantly from WT mice. WT mice infected with LAT(-) virus showed reduced latency, slower reactivation, and less T cell exhaustion than mice infected with LAT(+) virus. However, using different doses of each virus, latency levels, time of reactivation, and T cell exhaustion were similar between LAT(+) and LAT(-) viruses. These results suggest that the absence of IFNβ expression compensates for the function of LAT with regard to levels of latency, T cell exhaustion, and reactivation but does not affect viral and cellular transcripts during primary infection.

**IMPORTANCE:**

Interferon β (IFNβ) is a type I interferon that plays an important role in controlling primary herpes simplex virus type 1 (HSV-1) infection. To evaluate the importance of IFNβ on HSV-1 latency reactivation and its relationship to LAT, we infected IFNβ^-/-^ mice with LAT(+) and LAT(-) viruses. In the absence of IFNβ, latency levels in mice infected with LAT(-) virus were similar to those of mice infected with LAT(+) virus. The absence of IFNβ also reduced the time of reactivation in mice infected with LAT(-) virus to that of LAT(+) virus. Our results show a strong correlation between the functions of LAT and IFNβ during latent but not primary stages of HSV-1 infection.

## INTRODUCTION

The innate immune system is activated when an invading pathogen breaches the innate barrier and pathogen-associated molecular patterns on the pathogen surface or damage-associated molecular patterns on tissues damaged by pathogens are recognized by pattern-recognition receptors on innate immune cells (1). When innate immune cells patrolling the subepithelial tissue compartment sense an active pathogen, a series of events that induce interferons (IFNs), chemokines, and cytokines production and recruit inflammatory cells to the site of infection are triggered (2, 3). The IFN system is divided into types I, II, and III (4). Interferon types I and III play a pivotal role in pathogen clearance (5, 6). Type I IFNs include IFNα and IFNβ (7, 8), type II includes a single gene (IFNγ) (9), and type III includes three genes (10, 11). There is considerable interest in how IFNs control herpes simplex virus type 1 (HSV-1) infection because they are produced rapidly in response to viral infection and have antiviral activities.

IFNα/β generates strong antiviral responses to infection (12, 13). IFNα/β plays an important role in HSV infection *in vitro* and *in vivo* and is essential in controlling HSV-1 infection in the eyes of ocularly infected mice (14–16). However, HSV-1 can evade IFN responses through several mechanisms (17–22). Ectopic expression of IFNα or IFNβ markedly suppresses HSV-1 replication and spread and is associated with reduced lytic gene expression in infected mice (23, 24). IFNα/β genes bind to a heterodimer receptor, IFNAR1 (25). IFNAR1^-/-^ mice (also known as IFNαβR^-/-^ or CD118^-/-^) are highly susceptible to viral infections, including HSV-1 infection (19, 26–29). The ability of HSV-1 mutants to replicate in non-dividing cells in culture and *in vivo* was up to 1,000-fold higher in eyes and trigeminal ganglia (TG) of IFNAR1^-/-^ mice than in IFNγR^-/-^ mice (19, 30). Although they use the same receptor, IFNα subtypes and IFNβ have functional differences (31). Recently, we reported the initial generation and characterization of one of the 14 IFNα genes, IFNα2A (Roberson-A) (32). In contrast to highly susceptible IFNAR1^-/-^ mice and moderately susceptible IFNβ^-/-^ mice, IFNα2A^-/-^ mice are less susceptible to HSV-1 infection (32). Expressions of IFNα and IFNβ RNA were delayed in human (SK-N-SH) and mouse (neuro-2A) cells infected with LAT(+) (McKrae) virus compared with LAT(-) (dLAT2903) virus (33), suggesting a role for IFNβ in HSV-1 latency *in vivo*.

Most studies looking at the effects of type I IFN in HSV-1 infection involved primary infection using IFNAR1^-/-^ and IFNβ^-/-^ mice (19, 26–29). However, the effect of type I IFN receptor or IFNβ absence during latent HSV-1 infection has not been reported. Due to the crucial role of type I IFN in primary HSV-1 infection, we asked if IFNβ plays a role in HSV-1 latency reactivation by examining the effects of the absence of IFNβ on HSV-1 infectivity using IFNβ^-/-^ mice following infection with LAT(+) or LAT(-) viruses. In contrast to WT mice, we found that levels of latency in TG of IFNβ^-/-^ mice infected with different doses of LAT(+) or LAT(-) viruses were similar and not dose dependent. Also, in contrast to WT mice, time of reactivation and exhaustion levels were similar in LAT(+) and LAT(-) viruses. However, IFNβ^-/-^ mice were more susceptible to infection and had more eye disease than WT control mice despite having similar levels of virus replication in their eyes. As expected, the absence of LAT did not affect virus replication in the eye, viral transcripts, survival, or levels of eye disease in infected mice. These studies signify the key roles of IFNβ in controlling HSV latency reactivation, T cell exhaustion, eye disease, and host survival. Therefore, abolishing IFNβ expression can overcome the absence of LAT in LAT(-) viruses to recover the latency-reactivation function of LAT in LAT(+) viruses.

## RESULTS

### Virus replication in mouse tears

**Fig 1:**
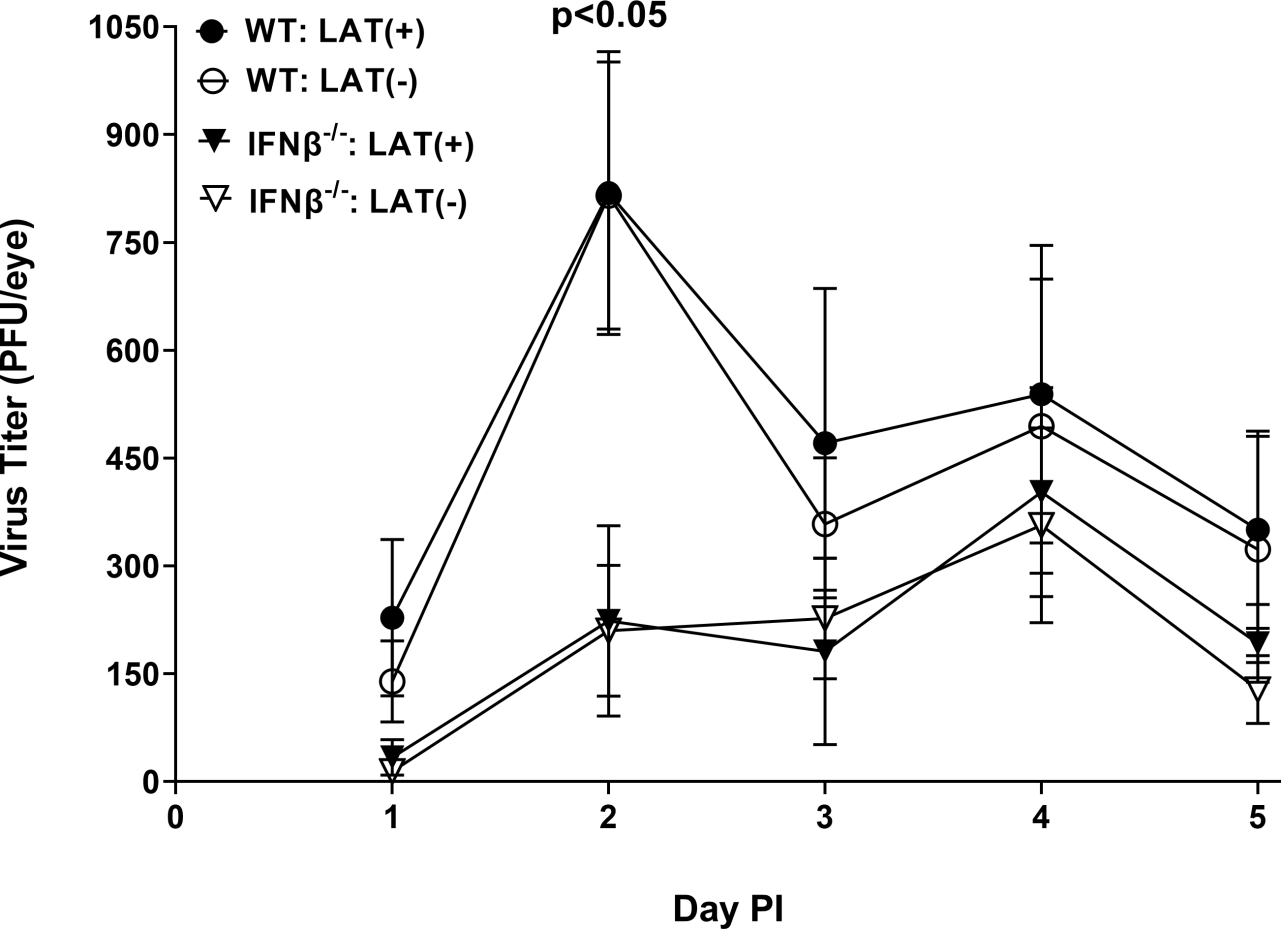
Virus titers in the eyes of infected mice. IFNβ^-/-^ and WT control mice were ocularly infected with 2 × 10^5^ PFU/eye of LAT(+) and LAT(-) viruses. Tear films were collected on days 1 to 5 PI, and virus titers were determined by standard plaque assay. Each point represents mean titers of 16 eyes for WT and 14 eyes for IFNα β^-/-^ mice from two separate experiments.

**Fig 2:**
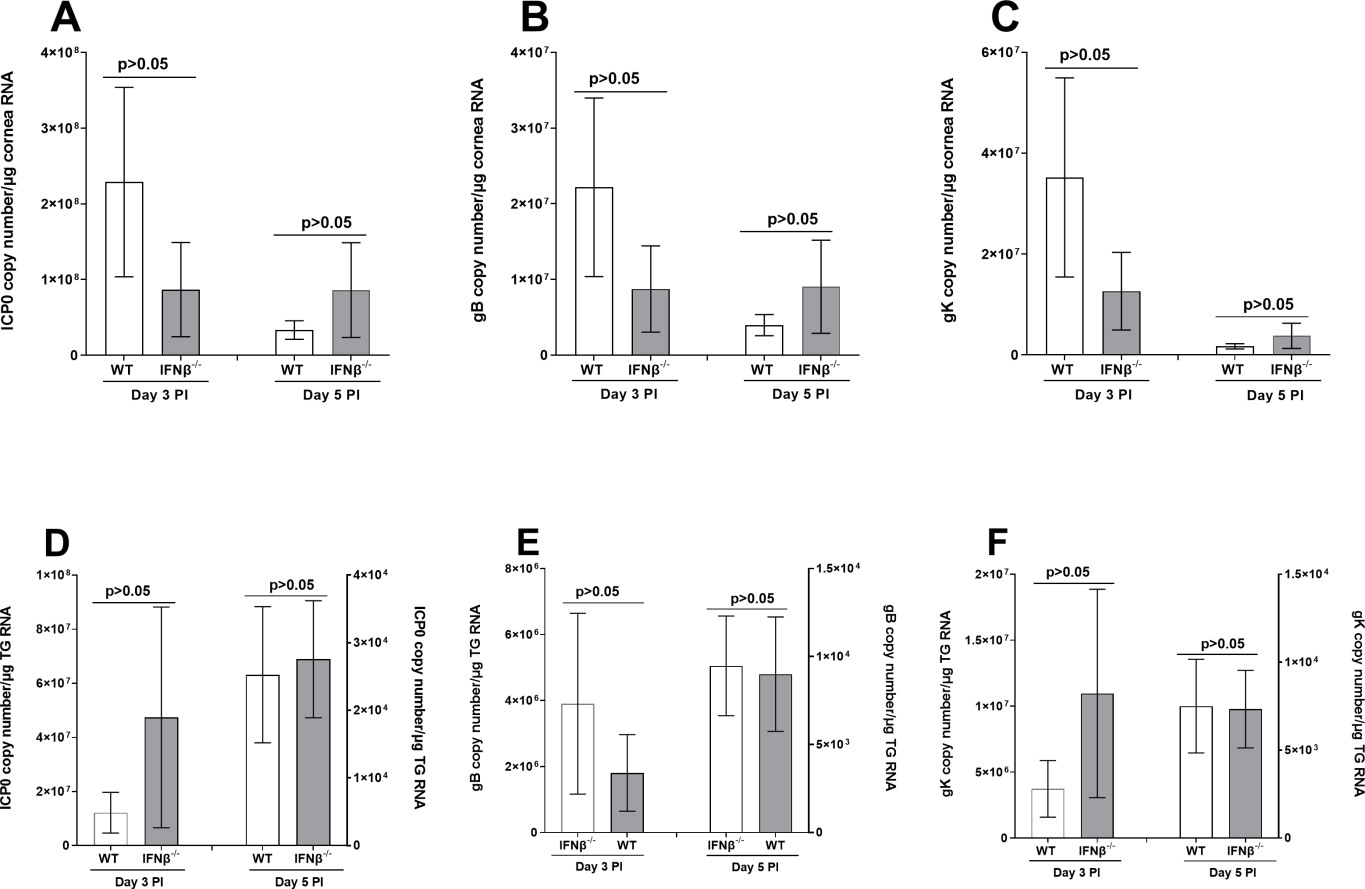
Viral gene expression in corneas and TG during primary mouse infection. IFNβ^-/-^ and WT mice were ocularly infected with 2 × 10^5^ PFU/eye of LAT(+) and LAT(-) viruses as in Fig. 1. Corneas (panels A, B, and C) and TG (panels D, E, and F) were harvested on days 3 and 5 PI. Total RNA was extracted, and ICP0 (panels A and D), gB (panels B and E), and gK (panels C and F) copy numbers were measured by qRT-PCR and normalized with GAPDH. Each point represents the mean ± SEM of 10 corneas and 10 TG for each day.

WT and IFNβ^-/-^ mice were ocularly infected with 2 × 10^5^ PFU/eye of LAT(+) or LAT(-) virus as described in Materials and Methods. Tear films from 20 eyes per group were collected on days 1 to 5 post-infection (PI), and the amount of virus was determined by standard plaque assays on rabbit skin (RS) cells (Fig. 1). On days 1, 3, 4, and 5 PI, virus replication was similar between the four groups (Fig. 2A; *P* > 0.05). On day 2 PI, IFNβ^-/-^ mice infected with LAT(+) or LAT(-) viruses had significantly lower virus titers than similarly infected WT mice (Fig. 1; *P* < 0.05, day 2 PI). Thus, the absence of IFNβ did not significantly affect virus replication in the eyes of IFNβ^-/-^ mice or WT mice except on day 2 PI. Similar to our previous studies (34, 35), the presence or absence of LAT did not affect virus replication in the eyes of infected mice.

### Expressions of viral and immune transcripts in corneas and TG of infected mice during primary ocular infection

**Fig 3:**
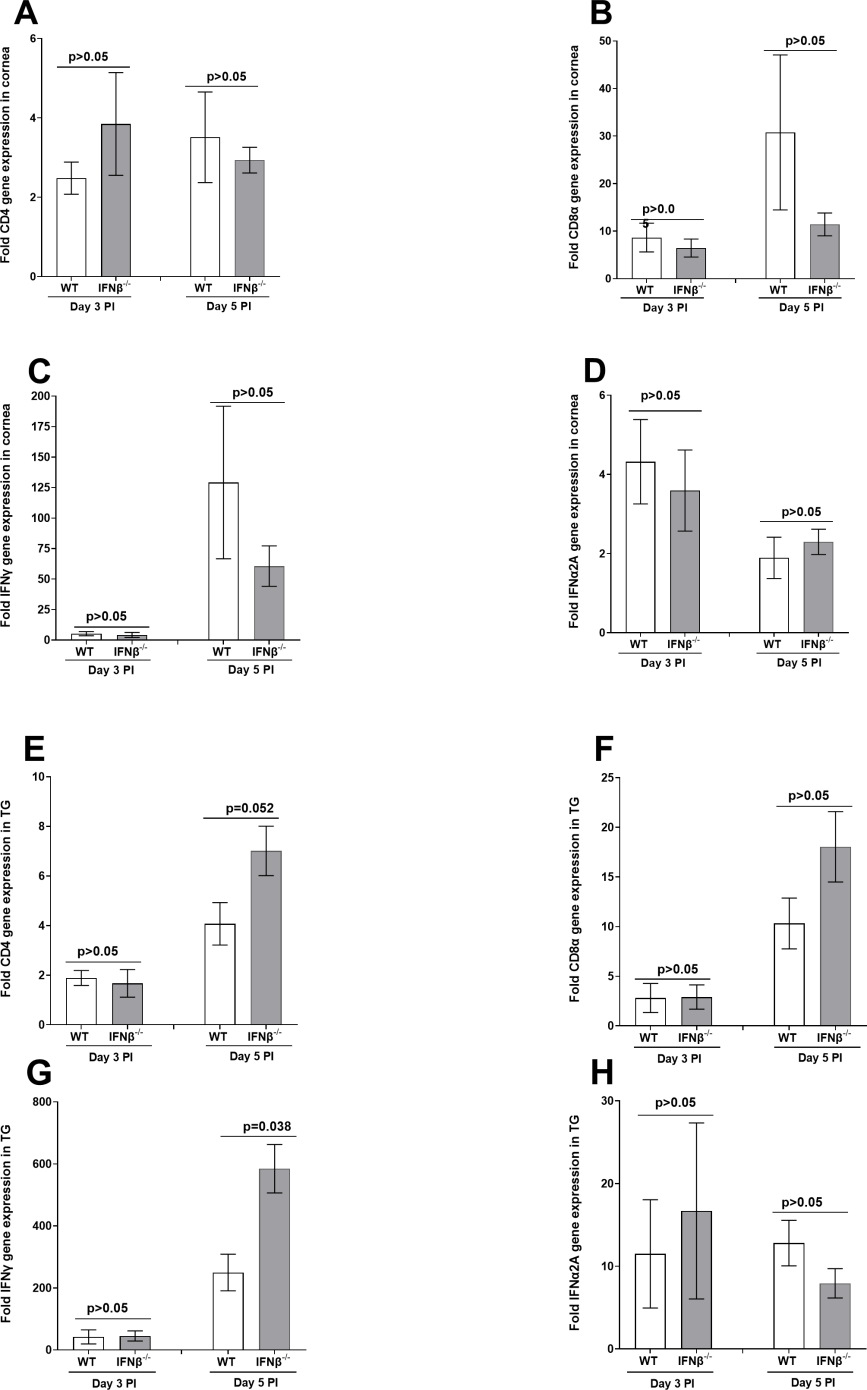
Effect of IFNβ absence on expression of immune genes during primary infection. RNA isolated on days 3 and 5 PI from corneas (panels A–D) and TG (panels E–H) of IFNβ^-/-^ (gray bars) and WT (open bars) infected mice as described in Fig. 2 above were used to measure the expression of CD4 (panels A and E), CD8α (panels B and F), IFNγ (panels C and G), and IFNα2A (panels D and H). qRT-PCR was performed as described above, and GAPDH was used as the endogenous control. Corneas and TG from uninfected IFNβ^-/-^ and WT mice were used for normalization. Each bar represents the mean fold change in expression of 10 corneas and 10 TG on each day.

In the absence of IFNβ, viral replication was similar in the eyes of infected mice and WT mice, except replication in IFNβ^-/-^ mice was less than in WT mice on day 2 PI (Fig. 1). We next asked whether the absence of IFNβ affects viral gene expression in corneas and TG of infected mice during primary infection. IFNβ^-/-^ and WT mice were infected as above. We found no statistically significant differences in the levels of each transcript in corneas of infected IFNβ^-/-^ and WT control mice on days 3 and 5 PI (Fig. 2A through C; ICP0, gB, and gK transcripts; *P* > 0.05, *t*-test). Similarly, no statistically significant differences in ICP0, gB, and gK expressions were detected on days 3 and 5 PI in TG of IFNβ^-/-^ and WT infected mice (Fig. 2D through F; ICP0, gB, and gK transcripts; *P* > 0.05, *t*-test). Although viral transcript levels were higher in the IFNβ^-/-^ group on day 3 PI, they were not statistically significant (Fig. 2D through F). Thus, the absence of IFNβ did not affect the expression of three different viral gene classes during primary infection, consistent with our results (Fig. 1) showing that the absence of IFNβ did not affect virus replication in the eyes of infected mice. However, our results with the expression of ICP0, gB, and gK transcripts in the cornea of infected mice on day 3 PI show a trend for reduction of these three transcripts between WT versus IFNβ^-/-^ mice. These findings, together with a significant decrease in virus titers in the eye of infected IFNβ^-/-^ mice on day 2 PI, may suggest a delay in virus replication early in the course of corneal infection, leading to a reduction in ICP0, gB, and gK expressions in the cornea of infected mice (Fig. 3).

The above results for the three tested viral transcripts suggested that IFNβ absence does not significantly alter virus replication in TG and corneas of infected and WT control mice. Therefore, we asked whether the absence of IFNβ in IFNβ ^-/-^ mice affected the expressions of CD4, CD8α, IFNγ, and IFNα2A transcripts in corneas and TG of infected mice on days 3 and 5 PI. RNA isolated from IFNβ^-/-^ and WT mice (see Fig. 2) was used to measure CD4, CD8α, IFNγ, and IFNα2A transcript levels in corneas and TG of infected mice by quantitative reverse transcription PCR (qRT-PCR). Results are presented as “fold” increase over baseline mRNA levels in corneas and TG of naive mice for each group. On days 3 and 5 PI, CD4, CD8α, IFNγ, and IFNα2A transcript levels were similar in corneas of infected mice with and without IFNβ expression (Fig. 3A through D; for all four transcripts; *P* > 0.05, *t*-test). Similarly, on day 3 PI, the CD4, CD8α, IFNγ, and IFNα2A expression in TG of WT and IFNβ^-/-^ infected mice was similar (Fig. 3E through H; for all four transcripts; *P* > 0.05, *t*-test, day 3 PI). Levels of IFNγ in TG of IFNβ^-/-^ infected mice on day 5 PI were significantly higher than in WT infected mice (Fig. 3G; IFNγ transcript; *P* = 0.038, *t*-test, day 5 PI). However, similar to day 3 results, CD4, CD8α, and IFNα2A transcript levels were similar in IFNβ^-/-^ infected and WT mice (Fig. 3E, F and H; for all three transcripts; *P* > 0.05, *t*-test, day 5 PI). Thus, except for IFNγ expression on day 5 PI, the absence of IFNβ did not affect the expression of the tested immune-related genes in either corneas or TG.

### Effect of IFNβ absence on HSV-1 latency in infected mice

**Fig 4:**
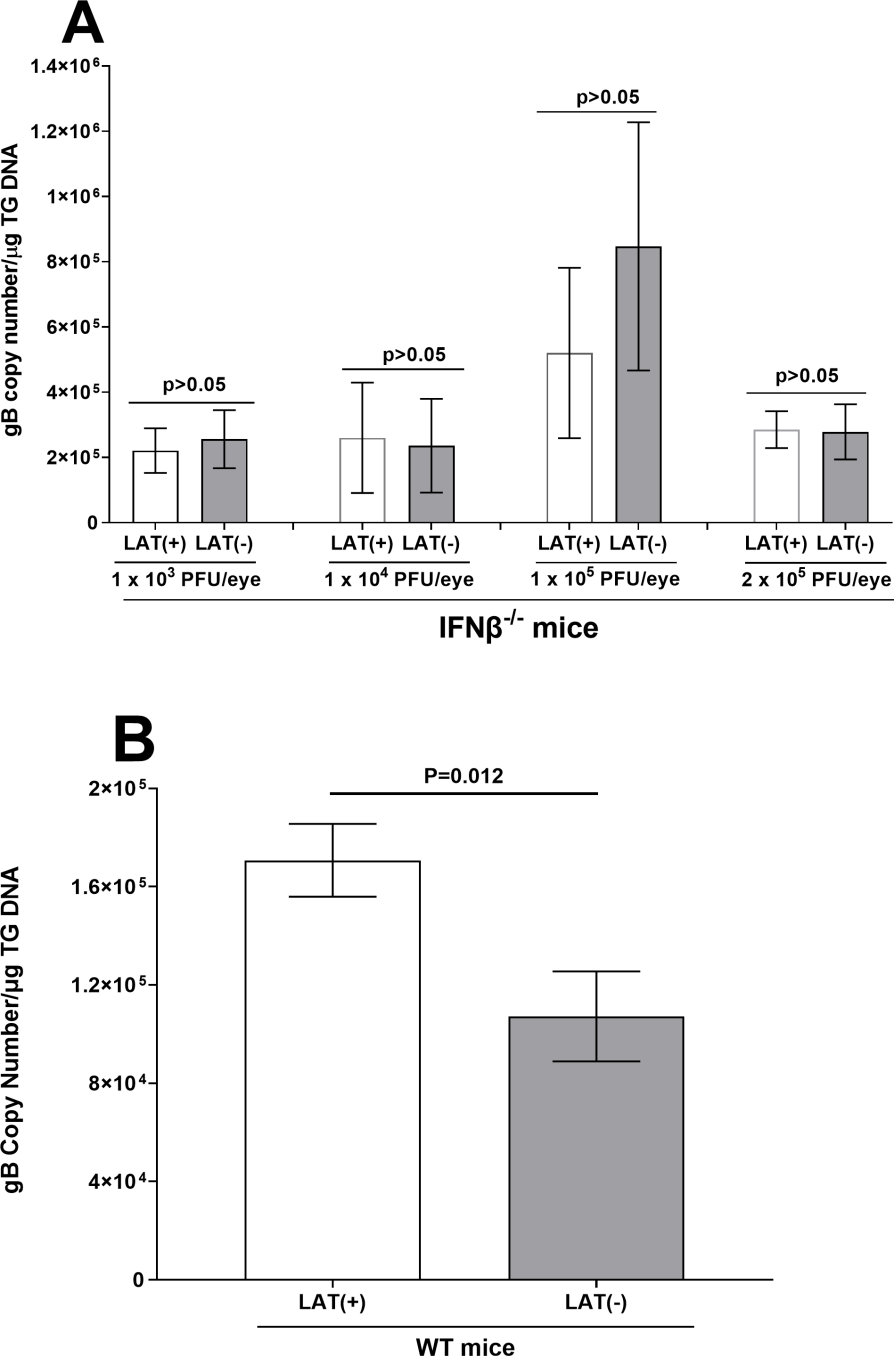
gB DNA levels in TG of latently infected mice. (**A**) Latency in IFNβ^-/-^ infected mice. IFNβ^-/-^ mice were ocularly infected with 1 × 10^3^, 1 × 10^4^, 1 × 10^5^, or 2 × 10^5^ PFU/eye of LAT(+) and LAT(-) viruses. TG from surviving mice was harvested on day 28 PI, and total TG DNA was isolated as described in Materials and Methods. gB DNA copy number was measured by qPCR using a standard curve generated with pAc-gB1 DNA. GAPDH expression was used to normalize relative levels of gB DNA expression. Each bar represents the mean ± SEM from 18, 12, 12, and 10 TG infected with 1 × 10^3^, 1 × 10^4^, 1 × 10^5^, and 2 × 10^5^ PFU/eye of LAT(+), respectively. Latency in mice infected with LAT(-) virus is based on 16 TG for 1 × 10^3^ PFU/eye, 14 TG for 1 × 10^4^ PFU/eye, 12 TG for 1 × 10^5^ PFU/eye, and 12 TG for 2 × 10^5^ PFU/eye; and (**B**) latency in WT infected mice. WT mice were ocularly infected with 2 × 10^5^ PFU/eye of LAT(+) and LAT(-) viruses. TG were isolated on day 28, DNA was extracted, and HSV-1 gB DNA levels were determined as above. Each bar represents the mean ± SEM from 46 TG for LAT(+) virus and 23 TG for LAT(-) virus from three independent experiments.

To investigate the effects of IFNβ absence on latency, we infected IFNβ^-/-^ mice with 1 × 10^3^, 1 × 10^4^, 1 × 10^5^, or 2 × 10^5^ PFU/eye of LAT(+) or LAT(-) virus (Fig. 4A). The data (gB copy number) from WT mice infected with 2 × 10^5^ PFU/eye of LAT(+) or LAT(-) virus was used as control (Fig. 4B). Individual TG were isolated on day 28 PI, total DNA [due to the absence of LAT in LAT(-) infected mice] was isolated, and TaqMan qPCR was performed to quantify viral gB DNA as described in Materials and Methods. The amount of latency in IFNβ^-/-^ mice infected with 1 × 10^3^, 1 × 10^4^, 1 × 10^5^, or 2 × 10^5^ PFU/eye of LAT(+) and LAT(-) viruses was similar to 2 × 10^5^ copy numbers per microgram of TG DNA (Fig. 4A; *P* > 0.05 for all four infection doses, Fisher's exact test). Consistent with previous reports from us and others (34–36), WT mice infected with 2 × 10^5^ PFU/eye of LAT(+) virus had significantly more gB DNA than WT mice infected with LAT(-) virus (Fig. 4B; *P* = 0.012). Latency levels in WT mice infected with LAT(+) virus were similar to those of IFNβ^-/-^ mice infected with either virus. In contrast to WT mice infected with LAT(+) virus, latency levels in WT mice infected with LAT(-) virus were lower than in IFNβ^-/-^ mice infected with either virus (compare Fig. 4A with Fig. 4B). These results suggest that, in the absence of IFNβ, latency levels are similar in LAT(+) and LAT(-) virus infections, and their levels are not affected by virus dose or absence of LAT. However, in the presence of LAT, latency levels are not affected by IFNβ absence, but IFNβ absence does affect latency levels in the absence of LAT.

### Effects of IFNβ absence on eye disease in infected mice

**Fig 5:**
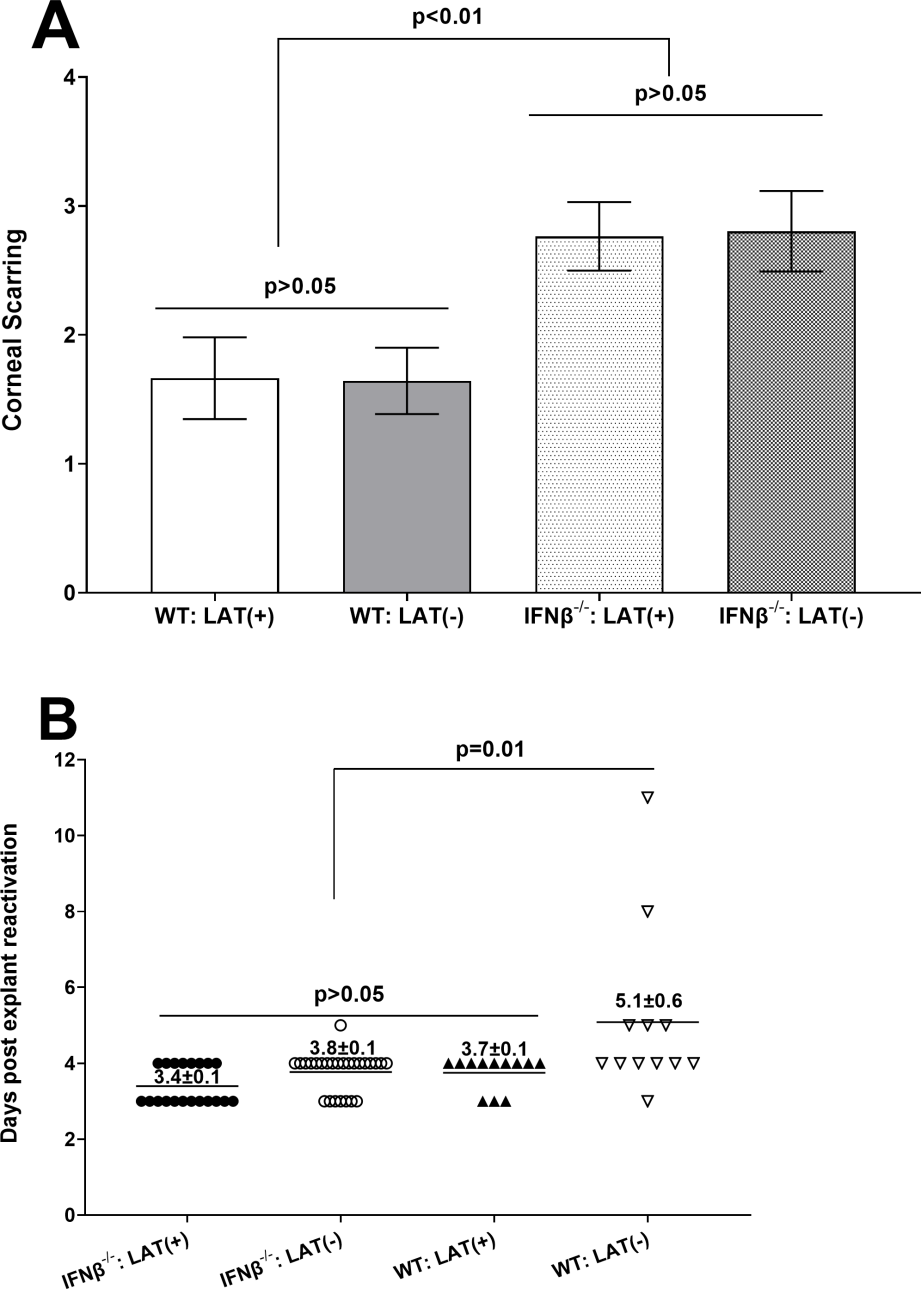
Effects of IFNβ absence on CS and explant reactivation. (**A**) CS in infected mice. Severity of CS on surviving mice infected with 2 × 10^5^ PFU/eye of LAT(+) and LAT(-) viruses was determined on day 28 PI as described in Materials and Methods. Each bar represents the mean ± SEM from 36 eyes [IFNβ^-/-^, LAT(+)], 26 eyes [IFNβ^-/-^, LAT(-)], 30 eyes [WT, LAT(+)], and 26 eyes [WT, LAT(-)]. (**B**) Virus reactivation in infected mice. IFNβ^-/-^ and WT control mice were ocularly infected with 1 × 10^5^ PFU/eye of LAT(+) and LAT(-) viruses as above. TG from infected mice were harvested on day 28 PI, and an explant reactivation assay was performed. Data points indicate the day of virus reactivation. Each point is the mean ± SEM from 20 TG for IFNβ^-/-^ mice infected with LAT(+) virus, 26 TG for IFNβ^-/-^ mice infected with LAT(-) virus, and 12 TG for WT groups infected with LAT(+) and LAT(-) viruses. Data for IFNβ^-/-^ infected mice are based on two independent experiments, while data from WT infected mice are based on one experiment.

A total of 44 and 49 IFNβ^-/-^ mice were infected with 2 × 10^5^ PFU/eye of LAT(+) or LAT(-) virus, respectively. Similarly, 18 and 28 WT mice were infected with LAT(+) or LAT(-) virus, respectively. Corneal scarring (CS) in surviving mice was measured on day 28 PI. Upon examining eyes for CS, we observed significantly more CS in IFNβ^-/-^ mice infected with virus than in WT control mice (Fig. 5A; *P* = 0.02). However, CS levels were similar in WT mice infected with either virus (Fig. 5A; WT, *P* > 0.05) and in IFNβ^-/-^ mice infected with virus (Fig. 5A; IFNβ^-/-^, *P* > 0.05). Thus, blocking IFNβ signaling increased CS over that seen in WT mice but not between LAT(+) or LAT(-) viruses. This increased CS did not depend on virus load in the eye since virus replication in the eye of infected mice, and viral transcripts in corneas and TG of infected mice on days 3 and 5 were similar (Fig. 1 and 2).

### Effects of IFNβ absence on reactivation in infected mice

In contrast to differing latency levels in TG of WT mice infected with LAT(+) and LAT(-) viruses (Fig. 4B, above), no differences in levels of gB DNA were detected in TG of IFNβ^-/-^ mice infected with four different doses of LAT(+) and LAT(-) viruses on day 28 PI (Fig. 4A). LAT is important for the high wt. rate of *in vivo* spontaneous (34) and induced (37) reactivation from latency in rabbits as well as higher reactivation in explant mouse cultures (35, 38, 39). Therefore, we asked whether the similarity in gB DNA levels in LAT(+) and LAT(-) infected IFNβ^-/-^ mice may also affect the time of reactivation. To test this and to increase the survival rate of infected mice, we infected IFNβ^-/-^ and WT mice with 1 × 10^5^ PFU/eye of LAT(+) and LAT(-) viruses rather than 2 × 10^5^ PFU/eye of each virus. TG were harvested from the four mouse groups on day 28 PI, and kinetics of virus reactivation were measured in explanted TG. The average reactivation time for IFNβ^-/-^ mice infected with LAT(+) virus was 3.4 ± 0.1 days, 3.8 ± 0.1 days for IFNβ^-/-^ mice infected with LAT(-) virus, and 3.7 ± 0.1 days for WT mice infected with LAT(+) virus, with no differences detected among the three groups (Fig. 5B; *P* > 0.05). In contrast, the average for WT mice infected with LAT(-) virus was 5.1 ± 0.6 days, which was significantly slower than the other three groups (Fig. 5B; *P* = 0.01). These results suggest that the absence of IFNβ immune signaling in IFNβ^-/-^ mice compensates for the absence of LAT in LAT(-) virus. Thus, the time of reactivation in LAT(-) infected mice was similar to LAT(+) virus. The similarity in time of reactivation between LAT(+) and LAT(-) viruses in IFNβ^-/-^ mice is independent of virus replication in the eyes or TG of infected mice during primary infection and is likely due to lack of IFNβ signaling during the latent state of latency.

### Effects of IFNβ absence on T cell exhaustion in TG of latently infected mice

**Fig 6:**
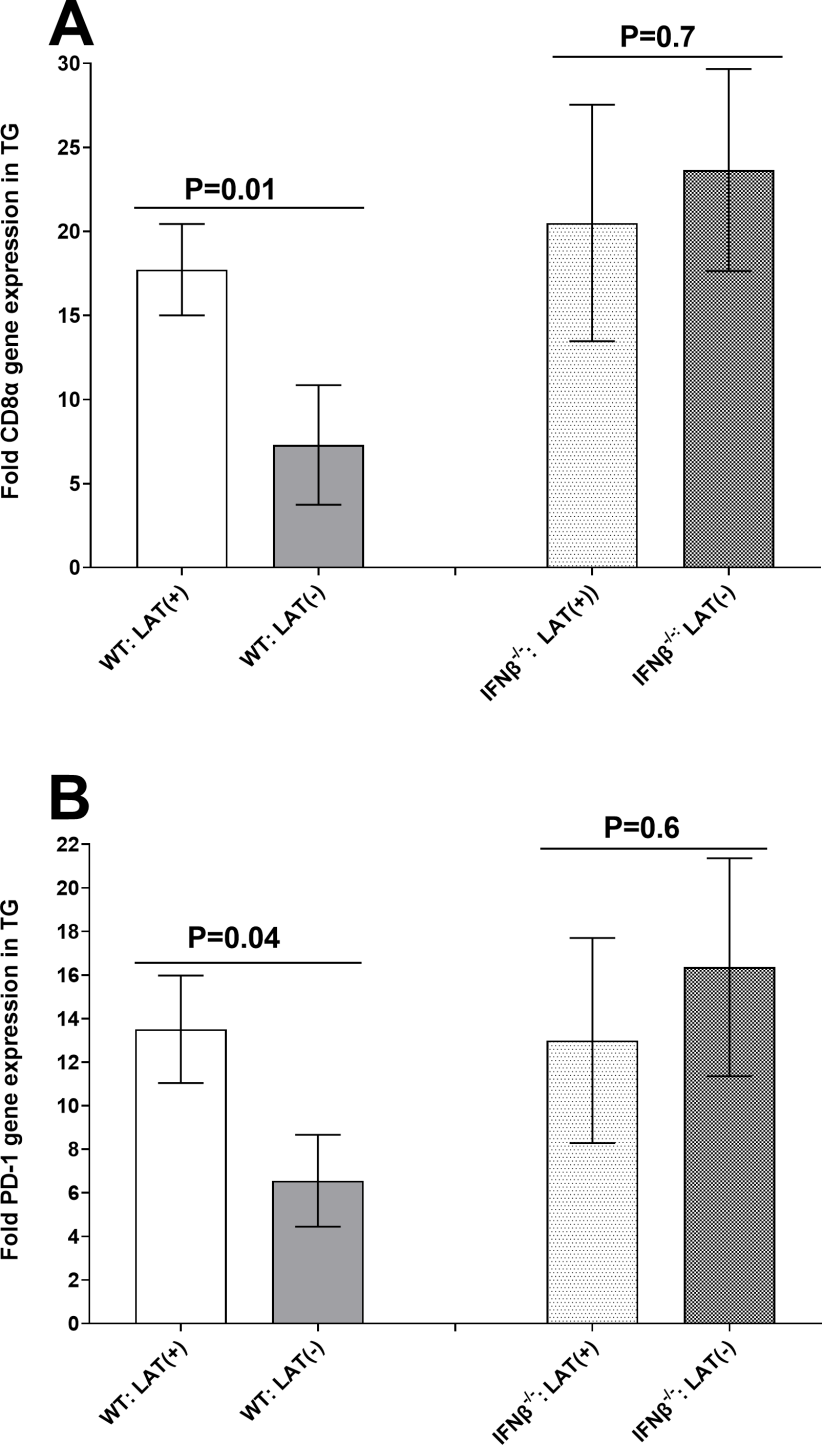
Effects of IFNβ absence on T cell exhaustion in TG of latently infected mice. IFNβ^-/-^ and WT mice were ocularly infected with 2 × 10^5^ PFU/eye of LAT(+) and LAT(-) viruses as above. TG from latently infected mice were individually isolated on day 28 PI, and qRT-PCR was performed using total RNA as described in Materials and Methods. CD8α (panel A) and PD-1 (panel B) expressions in naive mice were used as a baseline control to estimate relative expression of each transcript in TG of latently infected mice. GAPDH expression was used to normalize relative expression of each transcript. Each bar represents the mean ± SEM from 23 TG for WT mice infected with LAT(+), 12 TG for WT mice infected with LAT(-), 10 TG for IFNβ^-/-^ mice infected with LAT(+), and 14 TG for IFNβ^-/-^ mice infected with LAT(+) viruses.

Previous studies have reported lower latency levels and delayed time of reactivation in TG of WT mice infected with LAT(-) virus than in LAT(+) virus. In addition, LAT contributes to greater T cell exhaustion in TG of WT mice infected with LAT(+) virus than mice infected with LAT(-) virus (35, 40, 41). In contrast to WT mice, our results suggest that latency levels (Fig. 4B, above) and reactivation (Fig. 5B, above) were similar in LAT(+) and LAT(-) infected IFNβ^-/-^ mice. To determine how the absence of IFNβ expression affects T cell exhaustion, we infected WT and IFNβ^-/-^ mice with 2 × 10^5^ PFU/eye of LAT(+) or LAT(-) virus as described above. Relative levels of CD8 and PD-1 mRNAs were determined by qRT-PCR of total TG extracts. The results are presented as “fold” increase compared to baseline TG mRNA levels from uninfected naive mice (Fig. 6). In contrast to WT mice, levels of CD8 (Fig. 6A) and PD-1 (Fig. 6B) expression were significantly lower in TG of LAT(-) infected mice, while CD8 (Fig. 6A) and PD-1 (Fig. 6B) expression was similar in LAT(+) and LAT(-) infected IFNβ^-/-^ mice. These results suggest that the absence of IFNβ contributes to higher T cell exhaustion in TG of LAT(-) infected mice than in LAT(+) infected mice.

### Susceptibility of IFNβ^-/-^ mice following infection with LAT(+) and LAT(-) viruses

Groups of 6–49 IFNβ^-/-^ mice were infected ocularly in both eyes with 10-fold serial dilutions of LAT(+) and LAT(-) viruses ranging from 1 × 10^3^ to 2 × 10^5^ PFU/mouse eye. Control WT mice were infected with 1 × 10^5^ or 2 × 10^5^ PFU/eye of each virus. Survival of infected mice was monitored for 28 days. All IFNβ^-/-^ mice infected with 1 × 10^3^ and 1 × 10^4^ PFU/eye of each virus survived ocular infection (Table 1; *P* = 1). Sixteen of 29 (55%) of IFNβ^-/-^ mice survived infection with 1 × 10^5^ PFU/eye of LAT(+) virus, while 19 of 32 (59%) mice infected with LAT(-) virus survived infection. These differences were not statistically significant (Table 1; 1 × 10^5^, *P* = 0.92). Furthermore, 23 of 44 (52%) of IFNβ^-/-^ mice infected with 2 × 10^5^ PFU/eye of LAT(+) virus survived ocular infection, while 20 of 49 (41%) of mice infected with LAT(-) virus survived infection (Table 1; 2 × 10^5^, *P* = 0.34). In WT mice, six of the seven infected with 1 × 10^5^ PFU/eye of LAT(+) virus survived ocular infection, while six of eight infected with LAT(-) virus survived ocular infection (Table 1; WT, *P* = 0.9). Fifteen of 18 (83%) of WT mice infected with 2 × 10^5^ LAT(+) virus survived ocular infection, while 21 of 28 (75%) of mice infected with LAT(-) virus survived ocular infection (Table 1; WT, *P* = 0.41).

**TABLE 1:** Survival of IFNβ^-/-^ mice following ocular infection with different doses of LAT(+) (McKrae) and LAT(-) (dLAT2903) viruses^*a*^

	Surviving mice/total mice at dose of PFU/eye					
Virus	IFNβ^-/-^				WT	
	1 × 10^3^	1 × 10^4^	1 × 10^5^	2 × 10^5^	1 × 10^5^	2 × 10^5^
LAT(+) (McKrae)	9/9 (100%)	6/6 (100%)	16/29 (55%)	23/44 (52%)	6/7 (85%)	15/18 (83%)
LAT(-) (dLAT2903)	8/8 (100%)	7/7 (100%)	19/32 (59%)	20/49 (41)	6/8 (75%)	21/28 (75%)
*P* value	1	1	0.92	0.34	0.9	0.42

^*a*^ IFNβ^-/-^ mice were infected ocularly with the specified dose of each virus and survival was monitored for 28 days. Survival of IFNβ^-/-^ mice infected with 1 x 10^3^ and 1 x 10^4^ PFU/eye are based on one experiment, while survival of mice infected with 1 x 10^5^ and 2 x 10^5^ pfu/eye are based on four and six experiments, respectively. Survival of WT mice infected with 1 x 10^5^ and 2 x 10^5^ PFU/eye are based on one and three experiments, respectively.

In WT mice infected with 1 × 10^5^ PFU/eye of LAT(+) virus, six of seven (85%) mice survived ocular infection, while 16 of 29 (55%) of IFNβ^-/-^ mice infected with the same virus survived ocular infection, and these differences were statistically significant [Table 1; *P* < 0.05, 1 × 10^5^, LAT(+)]. Similar to mice infected with LAT(+) virus, six of eight (75%) WT mice infected with 1 × 10^5^ PFU/eye of LAT(-) virus survived ocular infection, while 19 of 32 (41%) of infected IFNβ^-/-^ mice survived ocular infection [Table 1; *P* < 0.05, 1 × 10^5^, LAT(-)]. WT mice infected with 2 × 10^5^ PFU/eye of LAT(+) virus: 15 of 18 (83%) mice survived ocular infection, while 23 of 44 (52%) of IFNβ^-/-^ mice infected with the same virus survived ocular infection, and these differences were statistically significant [Table 1; *P* = 0.03, 2 × 10^5^, LAT(+)]. Similar to LAT(+) infected mice, 21 of 28 (75%) of WT mice infected with 2 × 10^5^ PFU/eye of LAT(-) virus survived ocular infection, while 20 of 49 (41%) of infected IFNβ^-/-^ mice survived ocular infection [Table 1; *P* = 0.01, 2 × 10^5^, LAT(-)].

These results suggest that IFNβ^-/-^ mice are more refractory to HSV-1 infection than WT mice and that LAT(-) virus-infected IFNβ^-/-^ mice showed more virulence than LAT(+) infected mice, but these differences were not statistically significant. The higher susceptibility of IFNβ^-/-^ mice to LAT(-) infection may be due to the absence of LAT. We see similar trends in WT mice infected with LAT(-) or LAT(+) viruses, but those trends did not reach statistical significance.

## DISCUSSION

A primary characteristic of HSV-1 infection is the establishment of latency in humans and experimental animal models (42–46). In the latent phase of HSV-1 infection, expressions of more than 80 HSV-1 genes during lytic infection are drastically modified, and LAT is the only abundant transcript detected in TG of infected mice, rabbits, and humans during latency (37, 42, 43, 46–48). In a latently infected host, HSV-1 may reactivate, travel back to the original site of infection, and cause recurrent disease (49–53). With the use of LAT deletion mutants, multiple groups have reported that LAT enhances latency levels and slows time of reactivation in mice and rabbits (34, 37, 54–56). There are two copies of LAT per genome (46, 47), including an unstable 8.3 kb main transcript and a very stable 2 kb transcript derived by splicing from the 8.3 transcript (43, 48, 57, 58). LAT deletion mutants show significantly less reactivation in mice and rabbits (37, 54–56). As we have reported, the first 1.5 kb of the primary 8.3 kb LAT transcript is both necessary and sufficient to enhance reactivation in mouse and rabbit models (34, 56). We compared LAT(+) HSV-1 McKrae strain with LAT(-) dLAT2903 virus that, similar to reactivation in humans (59), shows significantly higher virus shedding in tears of infected rabbits (34). The explant-induced reactivation phenotype of dLAT2903 in mice is also slower than parental McKrae virus (35, 40). A deletion in dLAT2903 (−161 to +1667) prevents detectable LAT expression *in vitro* and *in vivo* (34), and reactivation of dLAT2903 is restored to WT levels when the first 1.5 kb of LAT promoter and coding sequence (nucleotides 1–1499) is inserted between UL37 and UL38 of dLAT2903 (56).

Four main characteristics of LAT(-) viruses are reduced latency levels, slower reactivation time, lower T cell exhaustion, and higher apoptosis in WT infected mice than in LAT(+) infected mice and rabbits (34, 35, 40, 41, 60). In contrast to WT mice infected with LAT(+) and LAT(-) viruses, in this study, we have shown that, in the absence of IFNβ, latency levels were similar in LAT(+) and LAT(-) viruses. Latency levels in IFNβ^-/-^ mice infected with different doses of LAT(+) and LAT(-) viruses were also similar. Furthermore, latency levels were similar in infected IFNβ^-/-^ mice and were significantly less than in WT infected mice irrespective of the virus dose. Lower latency levels in IFNβ^-/-^ infected mice than in WT mice occurred despite all four groups of infected mice having similar levels of ICP0, gB, and gK transcripts in their corneas and TG on days 3 and 5 PI. In WT mice, the reactivation time differed significantly in LAT(+) infected and LAT(-) infected mice (35, 40, 56). In contrast, reactivation times were similar in IFNβ^-/-^ mice infected with LAT(+) and LAT(-) viruses and similar to WT mice infected with LAT(+) virus. Reactivation was also faster in all three groups than in WT mice infected with LAT(-) virus. However, reactivation in WT mice infected with LAT(+) and LAT(-) viruses was similar to our previous reports (35, 40, 56).

Previously, we used LAT(+) and LAT(-) viruses to show that the presence of LAT leads to dysfunctional T cell responses in TG of latently infected WT mice (35). LAT expression and enhanced latency both correlated with increased CD8 and PD-1 mRNA levels in TG of latently infected mice. Our previous results in WT mice suggested that TG latently infected with LAT(+) virus contain more CD8^+^ T cells and more CD8^+^ T cells expressing the PD-1 exhaustion marker than TG latently infected with LAT(-) virus. In contrast to WT mice, the current study showed that IFNβ^-/-^ mice infected with LAT(+) and LAT(-) viruses have opposite effects on latency, reactivation, and T cell exhaustion in the absence of LAT in LAT(-) viruses. The anti-apoptotic function of LAT may regulate latency and reactivation (38, 60–64) or its ability to promote immune evasion by interfering with ICP0 and ICP4 expression in infected cells (65, 66). However, our results suggest the absence of LAT does not alter virus load in infected mice during primary infection. Rather, the absence of IFNβ compensates for LAT absence, causing IFNβ^-/-^ infected mice to behave similar to LAT(+) virus. LAT interferes with host IFNα/β expression in TG of infected mice, and this interference does not require other HSV-1 genes that inhibit IFNα/β signaling (67–72). Thus, our results confirm the previous study and emphasize the importance of IFNβ in HSV-1 latency reactivation and in T cell exhaustion, but not in primary infection. Previously, it was shown that the DNA-dependent activator of IFN regulatory factor (ZBP1) is required for IFNβ- and IFNγ-induced necroptosis (73). Since LAT interferes with apoptosis, in IFNβ^-/-^ mice, the lack of LAT's interference with the programmed cell death function of LAT and this role of LAT would be compensated by the absence of IFNβ, leading to reduced neuronal cell death. Therefore, IFNβ absence could inhibit virus-induced necroptosis, thus compensating for the cell death caused by the absence of LAT in LAT-minus virus.

Our results also show that the absence of IFNβ does not negatively impact primary infection on days 3 and 5 PI as determined by virus replication levels in the eyes of infected mice and by the lack of changes in ICP0, gB, and gK transcript levels in corneas and TG of infected mice. Thus, IFNβ absence did not enhance HSV-1 replication and spread in infected mouse eyes or TG. However, despite increased lytic gene expression in infected mice, the absence of IFNβ was associated with increased eye disease and decreased survival in infected mice. These changes were not affected by the presence or absence of LAT. The 14 IFNα subtypes and single IFNβ gene bind to a heterodimer receptor composed of IFNAR1 and IFNAR2 (25, 74). IFNAR1^-/-^ mice are more susceptible to HSV-1 infection (19, 28, 29, 75) than IFNβ^-/-^ mice, as we reported here. At a dose of 1 × 10^3^ PFU/eye, none of the infected IFNAR1^-/-^ mice survived ocular infection (data not shown), while 100% of infected IFNβ^-/-^ mice survived. Similar to this study, IFNβ^-/-^ mice were previously shown to be more susceptible to West Nile virus infection than WT mice (76). In contrast to our results, infected IFNβ^-/-^ mice had more virus replication in different tissues than WT control mice (76). IFNβ has anti-inflammatory properties, and treatment with recombinant IFNβ reduces inflammation and slows cartilage destruction in collagen-induced arthritis (77, 78). Inflammation is a protective response to infection that safeguards the body from side effects of infection and speeds the process of fixing damaged tissues (79). Consistent with the protective roles of inflammation in the healing process, our results confirm the importance of IFNβ anti-inflammatory properties in IFNβ^-/-^ mice by enhancing eye disease and reducing survival in infected mice. Thus, the absence of IFNβ in IFNβ^-/-^ mice may increase inflammation and disease, while blocking IFNβ can reduce latency in WT mice. Despite reduced latency in IFNβ^-/-^ infected mice in the presence or absence of LAT, the absence of IFNβ contributed to faster reactivation in infected mice. Recently, we were the first to construct and characterize a knockout mouse lacking IFNα2A (IFNα2A^-/-^) (32). In contrast to IFNβ^-/-^ mice, IFNα2A^-/-^ mice are more resistant to infection and did not have more eye disease than WT mice, but viral replication in the eyes of infected mice was similar to that in IFNβ^-/-^ mice. Similar to IFNβ^-/-^ mice in this study, we recently showed that the absence of IFNα2A significantly reduced latency. In contrast, IFNβ^-/-^ mice did not alter the time of reactivation (32). As reported here, the absence of IFNβ did not affect IFNα2A expression, while the absence of IFNα2A expression did affect IFNβ^-/-^ expression (32). In contrast to IFNβ, IFNα subtypes do not have known anti-inflammatory properties. Thus, the anti-inflammatory properties of IFNβ using IFNβ^-/-^ mice differ in primary and latent infections. During primary infection, IFNβ does not affect viral or cellular properties of infected mice in WT control mice but does affect survival, eye disease, latency, and reactivation in infected mice.

In conclusion, the results of this study show that the absence of IFNβ did not affect viral and cellular transcript levels during primary infection but did exacerbate CS in infected mice. However, the absence of IFNβ in LAT(-) virus altered levels of latency, reactivation, and T cell exhaustion similar to that of LAT(+) virus. Our results also suggest that latency reactivation and CD8 exhaustion functions of LAT were compensated by IFNβ absence, confirming that IFNβ plays a critical role in LAT function. Thus, IFNβ directly affects levels of HSV-1 latency reactivation and T cell exhaustion in WT mice.

## MATERIALS AND METHODS

### Mice, virus, and cells

C57BL/6 WT mice (6–8 weeks old) and IFNβ^-/-^ mice in C57BL/6 background were purchased from The Jackson Laboratory (Bar Harbor, ME, USA). All mice were bred and maintained in Cedars-Sinai Medical Center pathogen-free animal facility. No differences were detected between male and female WT and IFNβ^-/-^ mice infected with McKrae and dLAT2903 viruses; thus, the data for both male and female mice for each group were combined. Plaque-purified HSV-1 McKrae wild type, LAT(+), and dLAT2903 [LAT(-)] strains were grown in rabbit skin (RS) cell monolayers in minimal essential medium containing 5% fetal calf serum, as described previously (34, 80). dLAT2903 was derived from HSV-1 strain McKrae and replicated similar to McKrae *in vitro* and *in vivo* except it is LAT-negative (34). RS cells were used to prepare virus stocks, culture mouse tear swabs, and determine growth kinetics.

### Ocular infection

IFNβ^-/-^ mice were infected via the ocular route with 1 × 10^3^, 1 × 10^4^, 1 × 10^5^, and 2 × 10^5^ PFU/eye of LAT(+) and LAT(-) viruses. WT control mice were infected similarly but with 1 × 10^5^ or 2 × 10^5^ PFU/eye of each virus. Viruses were administered as an eye drop in 2 µL of tissue culture medium without prior corneal scarification.

### Virus titration in tears of infected mice

Mice were ocularly infected with 2 × 10^5^ PFU/eye of LAT(+) and LAT(-) viruses. Tear films were collected from both eyes of 10 mice per group on days 1–5 post-infection (PI) using a Dacron-tipped swab (81). Each swab was placed in 0.5 mL of tissue culture medium, squeezed, and the amount of virus was determined by a standard plaque assay on RS cells.

### *In vitro* explant reactivation assay

Mice were ocularly infected with 1 × 10^5^ PFU/eye of each virus, and infected mice were sacrificed on day 28 PI. Individual TG were removed and cultured in tissue culture media as we described previously (82). Media aliquots were removed from each culture daily for up to 12 days and plated on indicator RS cells to detect the appearance of reactivated virus. The time at which reactivated virus first appeared in the explanted TG cultures was determined based on daily plating of media from the explanted TG cultures.

### Monitoring corneal scarring (CS)

The severity of CS lesions in surviving mouse corneas on day 28 PI was examined by slit lamp biomicroscopy in a blinded fashion. CS was scored on a scale of 0 to 4 (0 = no disease, 1 = mild hazing, 2 = moderate opacity, 3 = severe corneal opacity, but iris visible, 4 = opaque and corneal ulceration).

### DNA extraction and PCR analysis to determine latency levels in TG of latently infected mice

WT and IFNβ^-/-^ mice were ocularly infected with 1 × 10^3^, 1 × 10^4^, 1 × 10^5^, or 2 × 10^5^ PFU/eye of LAT(+) and LAT(-) viruses. TG from individual mice were isolated on day 28 PI, and DNA was extracted from homogenized individual TG using the commercially available DNeasy Blood & Tissue Kit (Qiagen, Stanford, CA) according to the manufacturer's instructions. PCR analyses were performed using gB specific primers (Forward - 5′-AACGCGACGCACATCAAG-3′; Reverse - 5′-CTGGTACGCGATCAGAAAGC-3′; and Probe - 5′-FAM-CAGCCGCAGTACTACC-3′). The amplicon length for this primer set is 72 bp. Relative copy numbers of gB DNA were calculated using standard curves generated from the plasmid pAc-gB1 (83). In all experiments, GAPDH was used to normalize transcripts.

### Gene expression during primary infection

WT and IFNβ^-/-^ mice were ocularly infected with 2 × 10^5^ PFU/eye of LAT(+) and LAT(-) viruses. Corneas and TG from individual mice were isolated on days 3 and 5 PI, immersed in TRIzol reagent, and stored at −80°C until processing. Corneas and TG from each mouse were processed for RNA extraction as described previously (84). Isolated total RNA was reverse transcribed using a high-capacity cDNA reverse transcription kit (Applied Biosystems, Foster City, CA) according to the manufacturer's instructions. Expression levels of gB, gK, ICP0, CD4, CD8α, PD-1, IFNγ, and IFNα2a transcripts were determined using custom-made TaqMan primer sets as follows: for gK, 5′-GGCCACCTACCTCTTGAACTAC-3′ (forward), 5′-CAGGCGGGTAATTTTCGTGTAG-3′ (reverse), and probe 5′-FAM-CAGGCCGCATCGTATC-3; and for ICP0, 5′-CGGACACGGAACTGTTCGA-3′ (forward), 5′-CGCCCCCGCAACTG-3′ (reverse), and probe 5′-FAM-CCCCATCCACGCCCTG-3′. For gB, TaqMan primer sets are described above in the section measuring the level of latency. Other TaqMan primers from Applied Biosystems used in this study include: CD4 (Mm00442754_m1), CD8α (Mm01182107_g1), PD-1 (Mm00435532_m1), IFNγ (Mm01168134_m1), and IFNa2A (Mm00833961_s1). GAPDH primers from Applied Biosystems (assay identifier [ID], m999999.15_G1) were used as an internal control.

In each experiment, estimated relative copy numbers of gB, gK, and ICP0 genes were calculated using standard curves generated from plasmids containing the gene of interest: pGem-gK (for gK) (85), pAc-gB1 (for gB) (83), and pcDNA-ICP0 (for ICP0) (86). Estimated relative copy numbers of gB, gK, and ICP0 expressions were calculated using standard curves generated from their respective plasmids. Briefly, plasmid DNA template was serially diluted 10-fold so that 1  µL contained from 10^3^ to 10^8^ copies of the desired gene. The diluted template was then amplified by TaqMan PCR with the same primer set as the test samples. Copy numbers for each reaction product were determined by comparing the normalized threshold cycle of each sample to the standard curve threshold cycle. The 2−ΔΔCT method was used to analyze fold change in expression of CD4, CD8α, IFNγ, and IFNα2a, by comparing fold change in gene expression to expression in uninfected controls in each group. Statistical differences were calculated by ΔCT value.

### Statistical analysis

Student's *t*-test, Fisher's exact test, and analysis of variance (ANOVA) test were performed using the Instat computer program (GraphPad, San Diego, CA). Results were considered statistically significant at a *P* value of <0.05. All experiments were repeated at least two times to ensure accuracy.

## References

[1] Matzinger P. 1994. Tolerance, danger, and the extended family. Annu Rev Immunol 12:991–1045. doi:10.1146/annurev.iy.12.040194.005015 8011301

[2] Biswas PS, Rouse BT. 2005. Early events in HSV keratitis—setting the stage for a blinding disease. Microbes Infect 7:799–810. doi:10.1016/j.micinf.2005.03.003 15857807

[3] Jamali A, Hu K, Sendra VG, Blanco T, Lopez MJ, Ortiz G, Qazi Y, Zheng L, Turhan A, Harris DL, Hamrah P. 2020. Characterization of resident corneal plasmacytoid dendritic cells and their pivotal role in herpes simplex keratitis. Cell Rep 32:108099. doi:10.1016/j.celrep.2020.108099 32877681 PMC7511260

[4] Stark GR, Kerr IM, Williams BR, Silverman RH, Schreiber RD. 1998. How cells respond to interferons. Annu Rev Biochem 67:227–264. doi:10.1146/annurev.biochem.67.1.227 9759489

[5] Yin Y, Favoreel HW. 2021. Herpesviruses and the type III interferon system. Virol Sin 36:577–587. doi:10.1007/s12250-020-00330-2 33400088 PMC8379308

[6] Antony F, Pundkar C, Sandey M, Jaiswal AK, Mishra A, Kumar A, Channappanavar R, Suryawanshi A. 2021. IFN-λ regulates neutrophil biology to suppress inflammation in herpes simplex virus-1-induced corneal immunopathology. J Immunol 206:1866–1877. doi:10.4049/jimmunol.2000979 33811102

[7] Pestka S, Krause CD, Walter MR. 2004. Interferons, interferon-like cytokines, and their receptors. Immunol Rev 202:8–32. doi:10.1111/j.0105-2896.2004.00204.x 15546383

[8] de Weerd NA, Samarajiwa SA, Hertzog PJ. 2007. Type I interferon receptors: biochemistry and biological functions. J Biol Chem 282:20053–20057. doi:10.1074/jbc.R700006200 17502368

[9] Stetson DB, Medzhitov R. 2006. Type I interferons in host defense. Immunity 25:373–381. doi:10.1016/j.immuni.2006.08.007 16979569

[10] Ank N, West H, Paludan SR. 2006. IFN-λ: novel antiviral cytokines. J Interferon Cytokine Res 26:373–379. doi:10.1089/jir.2006.26.373 16734557

[11] Uzé G, Monneron D. 2007. IL-28 and IL-29: newcomers to the interferon family. Biochimie 89:729–734. doi:10.1016/j.biochi.2007.01.008 17367910

[12] Theofilopoulos AN, Baccala R, Beutler B, Kono DH. 2005. Type I interferons (α/β) in immunity and autoimmunity. Annu Rev Immunol 23:307–336. doi:10.1146/annurev.immunol.23.021704.115843 15771573

[13] McNab F, Mayer-Barber K, Sher A, Wack A, O’Garra A. 2015. Type I interferons in infectious disease. Nat Rev Immunol 15:87–103. doi:10.1038/nri3787 25614319 PMC7162685

[14] Hendricks RL, Weber PC, Taylor JL, Koumbis A, Tumpey TM, Glorioso JC. 1991. Endogenously produced interferon alpha protects mice from herpes simplex virus type 1 corneal disease. J Gen Virol 72 (Pt 7):1601–1610. doi:10.1099/0022-1317-72-7-1601 1649898

[15] Su YH, Oakes JE, Lausch RN. 1990. Ocular avirulence of a herpes simplex virus type 1 strain is associated with heightened sensitivity to α/β interferon. J Virol 64:2187–2192. doi:10.1128/JVI.64.5.2187-2192.1990 2157880 PMC249378

[16] Koujah L, Suryawanshi RK, Shukla D. 2019. Pathological processes activated by herpes simplex virus-1 (HSV-1) infection in the cornea. Cell Mol Life Sci 76:405–419. doi:10.1007/s00018-018-2938-1 30327839 PMC6349487

[17] Mittnacht S, Straub P, Kirchner H, Jacobsen H. 1988. Interferon treatment inhibits onset of herpes simplex virus immediate-early transcription. Virology (Auckl) 164:201–210. doi:10.1016/0042-6822(88)90637-x 2834869

[18] Mikloska Z, Cunningham AL. 2001. Alpha and gamma interferons inhibit herpes simplex virus type 1 infection and spread in epidermal cells after axonal transmission. J Virol 75:11821–11826. doi:10.1128/JVI.75.23.11821-11826.2001 11689663 PMC114768

[19] Leib DA, Harrison TE, Laslo KM, Machalek MA, Moorman NJ, Virgin HW. 1999. Interferons regulate the phenotype of wild-type and mutant herpes simplex viruses in vivo. J Exp Med 189:663–672. doi:10.1084/jem.189.4.663 9989981 PMC2192939

[20] De Regge N, Van Opdenbosch N, Nauwynck HJ, Efstathiou S, Favoreel HW. 2010. Interferon alpha induces establishment of alphaherpesvirus latency in sensory neurons in vitro. PLoS One 5:e13076. doi:10.1371/journal.pone.0013076 20927329 PMC2947521

[21] Mossman KL, Macgregor PF, Rozmus JJ, Goryachev AB, Edwards AM, Smiley JR. 2001. Herpes simplex virus triggers and then disarms a host antiviral response. J Virol 75:750–758. doi:10.1128/JVI.75.2.750-758.2001 11134288 PMC113971

[22] Leib DA. 2002. Counteraction of interferon-induced antiviral responses by herpes simplex viruses. Curr Top Microbiol Immunol 269:171–185. doi:10.1007/978-3-642-59421-2_11 12224508

[23] Noisakran S, Campbell IL, Carr DJ. 2000. IFN-alpha1 plasmid construct affords protection against HSV-1 infection in transfected L929 fibroblasts. J Interferon Cytokine Res 20:107–115. doi:10.1089/107999000312784 10670657

[24] Noisakran S, Carr DJ. 2000. Plasmid DNA encoding IFN-α1 antagonizes herpes simplex virus type 1 ocular infection through CD4^+^ and CD8^+^ T lymphocytes. J Immunol 164:6435–6443. doi:10.4049/jimmunol.164.12.6435 10843699

[25] Brierley MM, Fish EN. 2002. Review: IFN-α/β receptor interactions to biologic outcomes: understanding the circuitry. J Interferon Cytokine Res 22:835–845. doi:10.1089/107999002760274845 12396722

[26] Hwang SY, Hertzog PJ, Holland KA, Sumarsono SH, Tymms MJ, Hamilton JA, Whitty G, Bertoncello I, Kola I. 1995. A null mutation in the gene encoding a type I interferon receptor component eliminates antiproliferative and antiviral responses to interferons alpha and beta and alters macrophage responses. Proc Natl Acad Sci U S A 92:11284–11288. doi:10.1073/pnas.92.24.11284 7479980 PMC40616

[27] Müller U, Steinhoff U, Reis LF, Hemmi S, Pavlovic J, Zinkernagel RM, Aguet M. 1994. Functional role of type I and type II interferons in antiviral defense. Science 264:1918–1921. doi:10.1126/science.8009221 8009221

[28] Conrady CD, Thapa M, Wuest T, Carr DJJ. 2009. Loss of mandibular lymph node integrity is associated with an increase in sensitivity to HSV-1 infection in CD118-deficient mice. J Immunol 182:3678–3687. doi:10.4049/jimmunol.0803878 19265146 PMC2652688

[29] Luker GD, Prior JL, Song J, Pica CM, Leib DA. 2003. Bioluminescence imaging reveals systemic dissemination of herpes simplex virus type 1 in the absence of interferon receptors. J Virol 77:11082–11093. doi:10.1128/jvi.77.20.11082-11093.2003 14512556 PMC224994

[30] Conrady CD, Jones H, Zheng M, Carr DJJ. 2011. A functional type I interferon pathway drives resistance to cornea herpes simplex virus type 1 infection by recruitment of leukocytes. J Biomed Res 25:111–119. doi:10.1016/s1674-8301(11)60014-6 21709805 PMC3119485

[31] Platanias LC, Uddin S, Colamonici OR. 1994. Tyrosine phosphorylation of the alpha and beta subunits of the type I interferon receptor. Interferon-beta selectively induces tyrosine phosphorylation of an alpha subunit-associated protein. J Biol Chem 269:17761–17764. doi:10.1016/S0021-9258(17)32371-2 8027027

[32] Wang S, Jaggi U, Katsumata M, Ghiasi H. 2024. The importance of IFNα2A (Roferon-A) in HSV-1 latency and T cell exhaustion in ocularly infected mice. PLoS Pathog 20:e1012612. doi:10.1371/journal.ppat.1012612 39352890 PMC11469491

[33] Peng W, Henderson G, Inman M, BenMohamed L, Perng GC, Wechsler SL, Jones C. 2005. The locus encompassing the latency-associated transcript of herpes simplex virus type 1 interferes with and delays interferon expression in productively infected neuroblastoma cells and trigeminal Ganglia of acutely infected mice. J Virol 79:6162–6171. doi:10.1128/JVI.79.10.6162-6171.2005 15858001 PMC1091686

[34] Perng GC, Dunkel EC, Geary PA, Slanina SM, Ghiasi H, Kaiwar R, Nesburn AB, Wechsler SL. 1994. The latency-associated transcript gene of herpes simplex virus type 1 (HSV-1) is required for efficient in vivo spontaneous reactivation of HSV-1 from latency. J Virol 68:8045–8055. doi:10.1128/JVI.68.12.8045-8055.1994 7966594 PMC237268

[35] Allen SJ, Hamrah P, Gate D, Mott KR, Mantopoulos D, Zheng L, Town T, Jones C, von Andrian UH, Freeman GJ, Sharpe AH, BenMohamed L, Ahmed R, Wechsler SL, Ghiasi H. 2011. The role of LAT in increased CD8^+^ T cell exhaustion in trigeminal ganglia of mice latently infected with herpes simplex virus 1. J Virol 85:4184–4197. doi:10.1128/JVI.02290-10 21307196 PMC3126262

[36] Sawtell NM. 1997. Comprehensive quantification of herpes simplex virus latency at the single-cell level. J Virol 71:5423–5431. doi:10.1128/JVI.71.7.5423-5431.1997 9188614 PMC191782

[37] Hill JM, Sedarati F, Javier RT, Wagner EK, Stevens JG. 1990. Herpes simplex virus latent phase transcription facilitates in vivo reactivation. Virology (Auckl) 174:117–125. doi:10.1016/0042-6822(90)90060-5 2152989

[38] Inman M, Perng GC, Henderson G, Ghiasi H, Nesburn AB, Wechsler SL, Jones C. 2001. Region of herpes simplex virus type 1 latency-associated transcript sufficient for wild-type spontaneous reactivation promotes cell survival in tissue culture. J Virol 75:3636–3646. doi:10.1128/JVI.75.8.3636-3646.2001 11264353 PMC114855

[39] Jones C. 2003. Herpes simplex virus type 1 and bovine herpesvirus 1 latency. Clin Microbiol Rev 16:79–95. doi:10.1128/CMR.16.1.79-95.2003 12525426 PMC145298

[40] Allen SJ, Rhode-Kurnow A, Mott KR, Jiang X, Carpenter D, Rodriguez-Barbosa JI, Jones C, Wechsler SL, Ware CF, Ghiasi H. 2014. Interactions between herpesvirus entry mediator (TNFRSF14) and latency-associated transcript during herpes simplex virus 1 latency. J Virol 88:1961–1971. doi:10.1128/JVI.02467-13 24307582 PMC3911542

[41] Chentoufi AA, Kritzer E, Tran MV, Dasgupta G, Lim CH, Yu DC, Afifi RE, Jiang X, Carpenter D, Osorio N, Hsiang C, Nesburn AB, Wechsler SL, BenMohamed L. 2011. The herpes simplex virus 1 latency-associated transcript promotes functional exhaustion of virus-specific CD8^+^ T cells in latently infected trigeminal ganglia: a novel immune evasion mechanism. J Virol 85:9127–9138. doi:10.1128/JVI.00587-11 21715478 PMC3165846

[42] Stevens JG. 1989. Human herpesviruses: a consideration of the latent state. Microbiol Rev 53:318–332. doi:10.1128/mr.53.3.318-332.1989 2552271 PMC372739

[43] Wechsler SL, Nesburn AB, Watson R, Slanina S, Ghiasi H. 1988. Fine mapping of the major latency-related RNA of herpes simplex virus type 1 in humans. J Gen Virol 69 (Pt 12):3101–3106. doi:10.1099/0022-1317-69-12-3101 2848928

[44] Fraser NW, Valyi-Nagy T. 1993. Viral, neuronal and immune factors which may influence herpes simplex virus (HSV) latency and reactivation. Microb Pathog 15:83–91. doi:10.1006/mpat.1993.1059 8255209

[45] Phelan D, Barrozo ER, Bloom DC. 2017. HSV1 latent transcription and non-coding RNA: a critical retrospective. J Neuroimmunol 308:65–101. doi:10.1016/j.jneuroim.2017.03.002 28363461

[46] Rock DL, Nesburn AB, Ghiasi H, Ong J, Lewis TL, Lokensgard JR, Wechsler SL. 1987. Detection of latency-related viral RNAs in trigeminal ganglia of rabbits latently infected with herpes simplex virus type 1. J Virol 61:3820–3826. doi:10.1128/jvi.61.12.3820-3826.1987 2824816 PMC255998

[47] McGeoch DJ, Dalrymple MA, Davison AJ, Dolan A, Frame MC, McNab D, Perry LJ, Scott JE, Taylor P. 1988. The complete DNA sequence of the long unique region in the genome of herpes simplex virus type 1. J Gen Virol 69 (Pt 7):1531–1574. doi:10.1099/0022-1317-69-7-1531 2839594

[48] Wechsler SL, Nesburn AB, Watson R, Slanina SM, Ghiasi H. 1988. Fine mapping of the latency-related gene of herpes simplex virus type 1: alternative splicing produces distinct latency-related RNAs containing open reading frames. J Virol 62:4051–4058. doi:10.1128/JVI.62.11.4051-4058.1988 2845123 PMC253835

[49] Barron BA, Gee L, Hauck WW, Kurinij N, Dawson CR, Jones DB, Wilhelmus KR, Kaufman HE, Sugar J, Hyndiuk RA. 1994. Herpetic Eye Disease Study. A controlled trial of oral acyclovir for herpes simplex stromal keratitis. Ophthalmology 101:1871–1882. doi:10.1016/s0161-6420(13)31155-5 7997323

[50] Wilhelmus KR, Dawson CR, Barron BA, Bacchetti P, Gee L, Jones DB, Kaufman HE, Sugar J, Hyndiuk RA, Laibson PR, Stulting RD, Asbell PA. 1996. Risk factors for herpes simplex virus epithelial keratitis recurring during treatment of stromal keratitis or iridocyclitis. Herpetic Eye Disease Study Group. Br J Ophthalmol 80:969–972. doi:10.1136/bjo.80.11.969 8976723 PMC505673

[51] Gordon YJ. 1990. Pathogenesis and latency of herpes simplex virus type 1 (HSV-1): an ophthalmologist’s view of the eye as a model for the study of the virus-host relationship. Adv Exp Med Biol 278:205–209. doi:10.1007/978-1-4684-5853-4_21 1963036

[52] Kaufman HE, Azcuy AM, Varnell ED, Sloop GD, Thompson HW, Hill JM. 2005. HSV-1 DNA in tears and saliva of normal adults. Invest Ophthalmol Vis Sci 46:241–247. doi:10.1167/iovs.04-0614 15623779 PMC1200985

[53] Steiner I. 1996. Human herpes viruses latent infection in the nervous system. Immunol Rev 152:157–173. doi:10.1111/j.1600-065x.1996.tb00915.x 8930672

[54] Leib DA, Bogard CL, Kosz-Vnenchak M, Hicks KA, Coen DM, Knipe DM, Schaffer PA. 1989. A deletion mutant of the latency-associated transcript of herpes simplex virus type 1 reactivates from the latent state with reduced frequency. J Virol 63:2893–2900. doi:10.1128/JVI.63.7.2893-2900.1989 2542601 PMC250836

[55] Sawtell NM, Thompson RL. 1992. Herpes simplex virus type 1 latency-associated transcription unit promotes anatomical site-dependent establishment and reactivation from latency. J Virol 66:2157–2169. doi:10.1128/JVI.66.4.2157-2169.1992 1312626 PMC289008

[56] Perng GC, Ghiasi H, Slanina SM, Nesburn AB, Wechsler SL. 1996. The spontaneous reactivation function of the herpes simplex virus type 1 LAT gene resides completely within the first 1.5 kilobases of the 8.3-kilobase primary transcript. J Virol 70:976–984. doi:10.1128/JVI.70.2.976-984.1996 8551638 PMC189902

[57] Wagner EK, Flanagan WM, Devi-Rao G, Zhang YF, Hill JM, Anderson KP, Stevens JG. 1988. The herpes simplex virus latency-associated transcript is spliced during the latent phase of infection. J Virol 62:4577–4585. doi:10.1128/JVI.62.12.4577-4585.1988 2846871 PMC253569

[58] Farrell MJ, Dobson AT, Feldman LT. 1991. Herpes simplex virus latency-associated transcript is a stable intron. Proc Natl Acad Sci U S A 88:790–794. doi:10.1073/pnas.88.3.790 1846963 PMC50899

[59] Perng GC, Jones C. 2010. Towards an understanding of the herpes simplex virus type 1 latency-reactivation cycle. Interdiscip Perspect Infect Dis 2010:262415. doi:10.1155/2010/262415 20169002 PMC2822239

[60] Perng GC, Jones C, Ciacci-Zanella J, Stone M, Henderson G, Yukht A, Slanina SM, Hofman FM, Ghiasi H, Nesburn AB, Wechsler SL. 2000. Virus-induced neuronal apoptosis blocked by the herpes simplex virus latency-associated transcript. Science 287:1500–1503. doi:10.1126/science.287.5457.1500 10688801

[61] Carpenter D, Hsiang C, Brown DJ, Jin L, Osorio N, BenMohamed L, Jones C, Wechsler SL. 2007. Stable cell lines expressing high levels of the herpes simplex virus type 1 LAT are refractory to caspase 3 activation and DNA laddering following cold shock induced apoptosis. Virology (Auckl) 369:12–18. doi:10.1016/j.virol.2007.07.023 PMC2276668 17727910

[62] Jin L, Peng W, Perng GC, Brick DJ, Nesburn AB, Jones C, Wechsler SL. 2003. Identification of herpes simplex virus type 1 latency-associated transcript sequences that both inhibit apoptosis and enhance the spontaneous reactivation phenotype. J Virol 77:6556–6561. doi:10.1128/jvi.77.11.6556-6561.2003 12743314 PMC155006

[63] Peng W, Jin L, Henderson G, Perng GC, Brick DJ, Nesburn AB, Wechsler SL, Jones C. 2004. Mapping herpes simplex virus type 1 latency-associated transcript sequences that protect from apoptosis mediated by a plasmid expressing caspase-8. J Neurovirol 10:260–265. doi:10.1080/13550280490468690 15371157

[64] Branco FJ, Fraser NW. 2005. Herpes simplex virus type 1 latency-associated transcript expression protects trigeminal ganglion neurons from apoptosis. J Virol 79:9019–9025. doi:10.1128/JVI.79.14.9019-9025.2005 15994795 PMC1168792

[65] Chen SH, Kramer MF, Schaffer PA, Coen DM. 1997. A viral function represses accumulation of transcripts from productive-cycle genes in mouse ganglia latently infected with herpes simplex virus. J Virol 71:5878–5884. doi:10.1128/JVI.71.8.5878-5884.1997 9223477 PMC191843

[66] Garber DA, Schaffer PA, Knipe DM. 1997. A LAT-associated function reduces productive-cycle gene expression during acute infection of murine sensory neurons with herpes simplex virus type 1. J Virol 71:5885–5893. doi:10.1128/JVI.71.8.5885-5893.1997 9223478 PMC191844

[67] Johnson KE, Song B, Knipe DM. 2008. Role for herpes simplex virus 1 ICP27 in the inhibition of type I interferon signaling. Virology (Auckl) 374:487–494. doi:10.1016/j.virol.2008.01.001 PMC2638114 18279905

[68] Mossman KL, Saffran HA, Smiley JR. 2000. Herpes simplex virus ICP0 mutants are hypersensitive to interferon. J Virol 74:2052–2056. doi:10.1128/jvi.74.4.2052-2056.2000 10644380 PMC111685

[69] Kew C, Lui P-Y, Chan C-P, Liu X, Au SWN, Mohr I, Jin D-Y, Kok K-H. 2013. Suppression of PACT-induced type I interferon production by herpes simplex virus 1 Us11 protein. J Virol 87:13141–13149. doi:10.1128/JVI.02564-13 24067967 PMC3838286

[70] Cotter CR, Nguyen ML, Yount JS, López CB, Blaho JA, Moran TM. 2010. The virion host shut-off (vhs) protein blocks a TLR-independent pathway of herpes simplex virus type 1 recognition in human and mouse dendritic cells. PLoS One 5:e8684. doi:10.1371/journal.pone.0008684 20174621 PMC2823768

[71] Wang S, Wang K, Lin R, Zheng C. 2013. Herpes simplex virus 1 serine/threonine kinase US3 hyperphosphorylates IRF3 and inhibits beta interferon production. J Virol 87:12814–12827. doi:10.1128/JVI.02355-13 24049179 PMC3838156

[72] Paladino P, Mossman KL. 2009. Mechanisms employed by herpes simplex virus 1 to inhibit the interferon response. J Interferon Cytokine Res 29:599–607. doi:10.1089/jir.2009.0074 19694546

[73] Yang D, Liang Y, Zhao S, Ding Y, Zhuang Q, Shi Q, Ai T, Wu SQ, Han J. 2020. ZBP1 mediates interferon-induced necroptosis. Cell Mol Immunol 17:356–368. doi:10.1038/s41423-019-0237-x 31076724 PMC7109092

[74] Thomas C, Moraga I, Levin D, Krutzik PO, Podoplelova Y, Trejo A, Lee C, Yarden G, Vleck SE, Glenn JS, Nolan GP, Piehler J, Schreiber G, Garcia KC. 2011. Structural linkage between ligand discrimination and receptor activation by type I interferons. Cell 146:621–632. doi:10.1016/j.cell.2011.06.048 21854986 PMC3166218

[75] Conrady CD, Halford WP, Carr DJJ. 2011. Loss of the type I interferon pathway increases vulnerability of mice to genital herpes simplex virus 2 infection. J Virol 85:1625–1633. doi:10.1128/JVI.01715-10 21147921 PMC3028887

[76] Lazear HM, Pinto AK, Vogt MR, Gale M, Diamond MS. 2011. Beta interferon controls West Nile virus infection and pathogenesis in mice. J Virol 85:7186–7194. doi:10.1128/JVI.00396-11 21543483 PMC3126609

[77] van Holten J, Pavelka K, Vencovsky J, Stahl H, Rozman B, Genovese M, Kivitz AJ, Alvaro J, Nuki G, Furst DE, Herrero-Beaumont G, McInnes IB, Musikic P, Tak PP. 2005. A multicentre, randomised, double blind, placebo controlled phase II study of subcutaneous interferon beta-1a in the treatment of patients with active rheumatoid arthritis. Ann Rheum Dis 64:64–69. doi:10.1136/ard.2003.020347 15242865 PMC1755211

[78] van Holten J, Reedquist K, Sattonet-Roche P, Smeets TJM, Plater-Zyberk C, Vervoordeldonk MJ, Tak PP. 2004. Treatment with recombinant interferon-beta reduces inflammation and slows cartilage destruction in the collagen-induced arthritis model of rheumatoid arthritis. Arthritis Res Ther 6:R239–R249. doi:10.1186/ar1165 15142270 PMC416442

[79] Medzhitov R. 2008. Origin and physiological roles of inflammation. Nature 454:428–435. doi:10.1038/nature07201 18650913

[80] Osorio Y, Ghiasi H. 2003. Comparison of adjuvant efficacy of herpes simplex virus type 1 recombinant viruses expressing TH1 and TH2 cytokine genes. J Virol 77:5774–5783. doi:10.1128/jvi.77.10.5774-5783.2003 12719570 PMC154018

[81] Ghiasi H, Bahri S, Nesburn AB, Wechsler SL. 1995. Protection against herpes simplex virus-induced eye disease after vaccination with seven individually expressed herpes simplex virus 1 glycoproteins. Invest Ophthalmol Vis Sci 36:1352–1360. 7775113

[82] Mott KR, Ghiasi H. 2008. Role of dendritic cells in enhancement of herpes simplex virus type 1 latency and reactivation in vaccinated mice. Clin Vaccine Immunol 15:1859–1867. doi:10.1128/CVI.00318-08 18971304 PMC2593179

[83] Ghiasi H, Kaiwar R, Nesburn AB, Wechsler SL. 1992. Expression of herpes simplex virus type 1 glycoprotein B in insect cells: initial analysis of its biochemical and immunological properties. Virus Res 22:25–39. doi:10.1016/0168-1702(92)90087-P 1311136

[84] Mott KR, Perng GC, Osorio Y, Kousoulas KG, Ghiasi H. 2007. A recombinant herpes simplex virus type 1 expressing two additional copies of gK is more pathogenic than wild-type virus in two different strains of mice. J Virol 81:12962–12972. doi:10.1128/JVI.01442-07 17898051 PMC2169076

[85] Ghiasi H, Slanina S, Nesburn AB, Wechsler SL. 1994. Characterization of baculovirus-expressed herpes simplex virus type 1 glycoprotein K. J Virol 68:2347–2354. doi:10.1128/JVI.68.4.2347-2354.1994 8139020 PMC236711

[86] Matundan H, Ghiasi H. 2019. Herpes simplex virus 1 ICP22 suppresses CD80 expression by murine dendritic cells. J Virol 93:e01803-18. doi:10.1128/JVI.01803-18 30404803 PMC6340034

